# Fidelity in plant hormone modifications catalyzed by Arabidopsis GH3 acyl acid amido synthetases

**DOI:** 10.1016/j.jbc.2024.107421

**Published:** 2024-05-28

**Authors:** Cynthia K. Holland, Joseph M. Jez

**Affiliations:** 1 Department of Biology, Williams College, Williamstown, Massachusetts; 2 Department of Biology, Washington University in St Louis, St Louis, Missouri

**Keywords:** *Arabidopsis thaliana*, auxin, jasmonate, phytohormone, proofreading, substrate selectivity

## Abstract

GRETCHEN HAGEN 3 (GH3) acyl acid amido synthetases conjugate amino acids to acyl acid hormones to either activate or inactivate the hormone molecule. The largest subgroup of GH3 proteins modify the growth-promoting hormone auxin (indole-3-acetic acid; IAA) with the second largest class activating the defense hormone jasmonic acid (JA). The two-step reaction mechanism of GH3 proteins provides a potential proofreading mechanism to ensure fidelity of hormone modification. Examining pyrophosphate release in the first-half reaction of Arabidopsis GH3 proteins that modify IAA (AtGH3.2/YDK2, AtGH3.5/WES1, AtGH3.17/VAS2), JA (AtGH3.11/JAR1), and other acyl acids (AtGH3.7, AtGH3.12/PBS3) indicates that acyl acid-AMP intermediates are hydrolyzed into acyl acid and AMP in the absence of the amino acid, a typical feature of pre-transfer editing mechanisms. Single-turnover kinetic analysis of AtGH3.2/YDK2 and AtGH3.5/WES1 shows that non-cognate acyl acid-adenylate intermediates are more rapidly hydrolyzed than the cognate IAA-adenylate. In contrast, AtGH3.11/JAR1 only adenylates JA, not IAA. While some of the auxin-conjugating GH3 proteins in Arabidopsis (*i.e.*, AtGH3.5/WES1) accept multiple acyl acid substrates, others, like AtGH3.2/YDK2, are specific for IAA; however, both these proteins share similar active site residues. Biochemical analysis of chimeric variants of AtGH3.2/YDK2 and AtGH3.5/WES1 indicates that the C-terminal domain contributes to selection of cognate acyl acid substrates. These findings suggest that the hydrolysis of non-cognate acyl acid-adenylate intermediates, or proofreading, proceeds *via* a slowed structural switch that provides a checkpoint for fidelity before the full reaction proceeds.

Plant responses to developmental cues and the environment are primarily meditated through the activity and concentration of various phytohormones, including auxins, jasmonates, and benzoates (1, 2, 3, 4). For example, the primary auxin, indole-3-acetic acid (IAA), is involved in cell growth and division, elongation, and proliferation, while the jasmonates are involved in pathogen responses, root growth, seed germination, and fertility (1, 2, 3). Fluctuations in phytohormone concentrations within the plant cell and modulations of the chemical structure of a hormone affect plant hormone receptor interactions to alter physiological responses (5, 6, 7, 8, 9, 10, 11, 12).

As part of the biochemical system that maintains phytohormones in active and inactive forms, the GRETCHEN HAGEN 3 (GH3) family of acyl acid amido synthetases (also known as GH3 proteins) are responsible for maintaining hormone levels in plants by conjugating acyl acid hormones to various amino acids (11, 12, 13, 14, 15, 16). The physiological role of the phytohormone-amino acid conjugate formed by a GH3 protein depends upon both the hormone and the amino acid. Conjugation of amino acids to IAA results in inactivation with aspartate- and glutamate-IAA conjugates leading to hormone degradation and alanine- and valine-IAA conjugates serving as hormone storage forms (2, 7, 10). In the case of jasmonic acid (JA), addition of isoleucine forms the bioactive jasmonate hormone (+)-7-*iso*-jasmonyl-L-isoleucine (JA-Ile) (9, 17, 18, 19). Recent studies expand the biochemical function of GH3 proteins to include the modification of the IAA precursor (and auxin) indole-3-butyric acid (IBA) to modulate auxin-linked growth effects (20, 21); the biosynthesis of salicylic acid (SA), a critical plant pathogen defense signal, in Arabidopsis *via* an isochorismate-glutamate conjugate pathway (22, 23, 24); and the modification of benzoic acid (BA) and phenylacetic acid (PAA) (25). BA is a precursor to many plant metabolites, including SA, aromatic cytokinins, ubiquinone, folic acids, and multiple specialized metabolites that attract pollinators and seed dispersers (26, 27, 28, 29, 30, 31). PAA is a non-indolic auxin that regulates the same signaling pathways as IAA, targets the auxin receptor, and is widely distributed in both vascular and nonvascular plants (32).

The GH3 family of acyl acid amido synthetases is widely distributed in plants with multiple isoforms found in various species (8, 10, 11, 13, 33, 34, 35). All plants examined to date encode at least one GH3 protein that catalyzes the formation of the bioactive jasmonate JA-Ile. In *Arabidopsis thaliana* (thale cress), AtGH3.11/JAR1 is essential for synthesis of JA-Ile and loss-of-function blocks JA-mediated responses, including defense against herbivores, fungi, and pathogens (8, 9, 36, 37). Each plant species examined to date also encodes multiple GH3 proteins that modify IAA and other related compounds, such as IBA, BA, and PAA. For example, AtGH3.2/YDK1 from Arabidopsis primarily conjugates aspartate to IAA (25). Arabidopsis *ydk 1-D* mutants are dwarfed with short hypocotyls in both light and dark growth conditions, which indicates that the *YDK1* gene negatively regulates auxin signaling (38). Similarly, AtGH3.17/VAS2 is specific for IAA and regulates local auxin levels to hypocotyls in response to changes in shade and temperature (39). In contrast to these two GH3 proteins, AtGH3.5/WES1 contributes to both IAA and SA responses in Arabidopsis and is implicated in SA-linked pathogen responses *in vivo* (40, 41, 42, 43) and conjugates aspartate to IAA, PAA, and BA with comparable catalytic efficiency *in vitro* (25).

GH3 proteins generate phytohormone-amino acid conjugates through a two-step reaction mechanism (Fig. 1) (8, 10, 14, 34, 44, 45). In the first half-reaction, ATP is used to adenylate the acyl acid substrate with the release of pyrophosphate. Next, the nucleophilic amine group of an amino acid displaces AMP to form an acyl acid-amino acid conjugate. X-ray crystal structures of multiple GH3 proteins from Arabidopsis and grape (*Vitus vinifera*) suggest that structural changes in the flexible C-terminal domain are linked to the two half-reactions (20, 21, 24, 25, 34, 46, 47, 48). GH3 proteins share a common tertiary structure consisting of a large N-terminal domain and a smaller C-terminal domain with the active site at the interface of the two domains. A flexible hinge loop connects the two domains and pivots the C-terminal domain by 180° during the reaction cycle (Fig. 1). In the open conformation, ATP, Mg^2^^+^, and the acyl acid substrate bind in the active site for the adenylation reaction with a solvent-accessible channel allowing pyrophosphate release. Upon adenylation and pyrophosphate release, the C-terminal domain rotates about the hinge loop. This closes the active site, opens a channel for amino acid substrate binding, and repositions residues for the transferase reaction (34, 46, 48).

**Figure 1:**
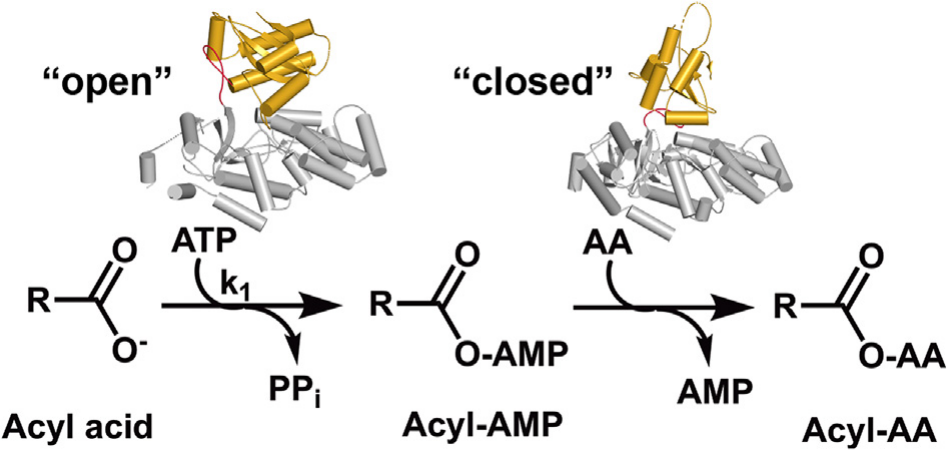
**Overall reaction catalyzed by GH3 proteins.** GH3 proteins act as acyl acid amido synthetases and catalyze a two-step reaction mechanism. In the first half-reaction, an acyl acid substrate, Mg^2+^, and ATP bind the open form of the protein to allow for adenylation of the acyl acid and formation of pyrophosphate. In the second half-reaction, the C-terminal domain (*gold*) pivots 180° to allow for amino acid binding and the transfer step, which results in formation of the acyl acid-amino acid conjugate and AMP.

Although biochemical and structural studies provide insight into the reaction chemistry and active site residues that delineate substrate specificity between JA-, IAA-, IBA-, and isochorismate-conjugating GH3 proteins (8, 9, 10, 19, 20, 21, 22, 23, 24, 25, 34, 35, 44, 45, 46, 47, 48), these same studies suggest an additional level of substrate selectivity. The x-ray crystal structures of AtGH3.5/WES1 (IAA/BA dual functional), AtGH3.11/JAR1 (JA), AtGH3.12/PBS3 (isochorismate), and AtGH3.15 (IBA) reveal significant amino acid differences in residues of the acyl acid binding site that leads to recognition of chemically distinct acyl acid substrates. Within the group of IAA-conjugating GH3 proteins from Arabidopsis, sequence comparisons show that the active site residues contacting IAA are conserved (25); however, each of these GH3 proteins has distinct acyl acid substrate profiles. For example, AtGH3.5/WES1 has comparable catalytic efficiencies (*k*_cat_/*K*_m_) for IAA (314 M^−1^ s^−1^), PAA (243 M^−1^ s^−1^), and BA (338 M^−1^ s^−1^); whereas AtGH3.2/YDK1 is more specific for IAA (556 M^−1^ s^−1^) than either PAA (125 M^−1^ s^−1^) or BA (77 M^−1^ s^−1^) (25). Biochemical comparisons of the IAA-conjugating GH3 proteins suggest that substrate selectivity is defined by more than the amino acid sequence of the acyl acid binding site.

Here we examine the contribution of the first-half reaction (*i.e.*, substrate adenylation) *versus* the overall reaction catalyzed by GH3 proteins to substrate selectivity. Pyrophosphate release assays that monitor the first-half reaction with and without amino acid of Arabidopsis GH3 proteins that modify IAA (AtGH3.2/YDK2, AtGH3.5/WES1, AtGH3.17/VAS2), JA (AtGH3.11/JAR1), and other acyl acids (AtGH3.7, AtGH3.12/PBS3) indicates that acyl acid-AMP intermediates are hydrolyzed into acyl acid and AMP in the absence of the amino acid, a typical feature of pre-transfer editing mechanisms. Using single-turnover kinetics, the rates of the first-half reaction for AtGH3.2/YDK1, AtGH3.5/WES1, and AtGH3.11/JAR1 were determined using various acyl acid substrates. The role of the C-terminal domain in the GH3 reaction is also examined using chimeric protein constructs of AtGH3.2/YDK1 and AtGH3.5/WES1. Overall, these experiments suggest that the kinetics of the first half-reaction and the C-terminal domain contributes to selection of cognate intermediates *versus* non-cognate intermediates in the synthesis of the appropriate final acyl acid-amino acid conjugate.

## Results

### Pyrophosphate release catalyzed by GH3 proteins

Previous studies of AtGH3.12/PBS3 indicated a substrate preference for chorismate (*k*_cat_/*K*_m_ = 4220 M^−1^ s^−1^) *versus* 4-HBA (*k*_cat_/*K*_m_ = 278 M^−1^ s^−1^) (24). Interestingly, analysis of pyrophosphate release catalyzed by AtGH3.12/PBS3 using 4-HBA and chorismate in either the absence or presence of glutamate showed a difference in the ratio of specific activities (24). The rates of pyrophosphate release using the non-physiological substrate 4-HBA as the acyl acid were similar with or without glutamate (Table 1). In contrast, there was a 26-fold higher activity with chorismate (as a substitute for unstable isochorismate) and glutamate as reactants compared to the reaction with chorismate alone (Table 1). Assays with BA yielded comparable (1.4-fold higher with glutamate) activities and assays with SA, a known inhibitor of AtGH3.12/PBS3, showed no detectable activity (Table 1). The pyrophosphate release activities of AtGH3.12/PBS3 with non-preferred substrates (*i.e.*, 4-HBA and BA) *versus* the slower rate in the first half-reaction with a preferred substrate (*i.e.*, chorismate) and rapid increased rate with amino acid suggested discrimination between cognate and non-cognate intermediates during the GH3 reaction sequence.

**Table 1:** Comparison of pyrophosphate release specific activities of Arabidopsis GH3 proteins for acyl acids in the absence and presence of conjugating amino acid

Protein	Amino acid	Acyl acid			
		BA	4-HBA	chorismate	SA
AtGH3.12/PBS3	−Glu	38 ± 3	120 ± 4^a^	24 ± 2^a^	ND^b^
	+Glu	57 ± 8	140 ± 2^a^	620 ± 14^a^	ND^b^
	ratio +Glu/−Glu	1.4	1.2	25.8	-
		BA	4-HBA	chorismate	
AtGH3.7	−Cys	14 ± 2	36 ± 5	44 ± 3	
	+Cys	16 ± 4	56 ± 5	401 ± 11	
	ratio +Cys/−Cys	1.1	1.6	9.1	
		IAA	JA		
AtGH3.11/JAR1	−Ile	ND^b^	4 ± 2		
	+Ile	ND^b^	44 ± 4		
	ratio +Ile/−Ile	-	11.0		
		IAA	IBA	PAA	BA
AtGH3.2/YDK2	−Asp	8 ± 4	15 ± 2	16 ± 3	10 ± 3
	+Asp	94 ± 4	29 ± 7	48 ± 5	24 ± 6
	ratio +Asp/−Asp	11.8	1.9	3.0	2.4
		IAA	IBA	PAA	BA
AtGH3.5/WES1	−Asp	24 ± 5	32 ± 4	38 ± 4	14 ± 1
	+Asp	121 ± 3	99 ± 2	133 ± 2	37 ± 2
	ratio +Asp/−Asp	5.0	3.1	3.5	2.6
		IAA	IBA	PAA	BA
AtGH3.17/VAS2	−Glu	5 ± 1	12 + 1	13 ± 2	2 ± 1
	+Glu	66 ± 9	12 ± 6	18 ± 1	2 ± 1
	ratio +Glu/−Glu	13.2	1.0	1.4	1.0

Pyrophosphate release assays were performed as described in the Experimental procedures. Specific activity (nmol min^−1^ mg protein^−1^) for each protein were determined in the absence and presence of preferred amino acid with selected acyl acids. The ratio of specific activity in the presence (+) and absence (−) of amino acid substrate is noted. Values shown are the average ± SE (*n* = 3).

a Specific activities for AtGH3.12/PBS3 for 4-HBA and chorismate were previously reported (24).

b No detectable activity.

To examine the difference between the first half-reaction (*i.e.*, adenylation step) and the full reaction (*i.e.*, transferase step), pyrophosphate release was measured for AtGH3.7, AtGH3.11/JAR1, AtGH3.2/YDK2, AtGH3.5/WES1, and AtGH3.17/VAS2 using different acyl acid substrates in either the absence or presence of an amino acid substrate (Table 1). Steady-state kinetic analyses of the full reaction catalyzed by each enzyme were previously reported (24, 25, 34). Pyrophosphate released during the first half-reaction accounts for acyl-AMP formation, while the full reaction includes the incorporation of an amino acid to form the final acyl acid-amino acid conjugate. Assays with AtGH3.7, which is in the same GH3 protein sub-group as AtGH3.12/PBS3 (24), showed a similar profile as AtGH3.12/PBS3 with an activity ratio in the presence of cysteine (the preferred amino acid substrate of AtGH3.7) 9-fold higher than without the amino acid and the activities with BA and 4-HBA showing comparable rates with or without cysteine (Table 1).

AtGH3.11/JAR1 is responsible for the formation of the bioactive jasmonate JA-Ile and is highly specific for the oxylipin JA as the acyl acid substrate (8, 9, 19, 34). This protein did not exhibit a detectable pyrophosphate release activity with a non-substrate (*i.e.*, IAA) but has an 11-fold higher activity with isoleucine compared to assays without the amino acid (Table 1).

In Arabidopsis, multiple GH3 proteins are associated with conjugation of the major phytohormone IAA with either aspartate or glutamate (10, 11, 16, 25, 34, 38, 39, 40, 41, 42, 43); however, different GH3 proteins display varied acyl acid profiles from highly specific for IAA (AtGH3.2/YDK2 and AtGH3.17/VAS2) to relaxed substrate selectivity (AtGH3.5/WES1) (25, 39). These three enzymes were assayed for pyrophosphate release using IAA, IBA, PAA, and BA as acyl acid substrates with and without aspartate (AtGH3.2/YDK2 and AtGH3.5/WES1) or glutamate (AtGH3.17/VAS2) (Table 1). Both AtGH3.2 YDK2 and AtGH3.17/VAS2 had higher specific activities with IAA in the presence of aspartate (∼12-fold) and glutamate (∼13-fold), respectively, compared to the lower ratios with IBA, PAA, and BA. In comparison, AtGH3.5/WES1 exhibits a modestly higher activity with IAA and aspartate (5-fold) *versus* the other acyl acids (2.6–3.5-fold).

### TLC analysis of GH3 protein-catalyzed reactions

**Figure 2:**
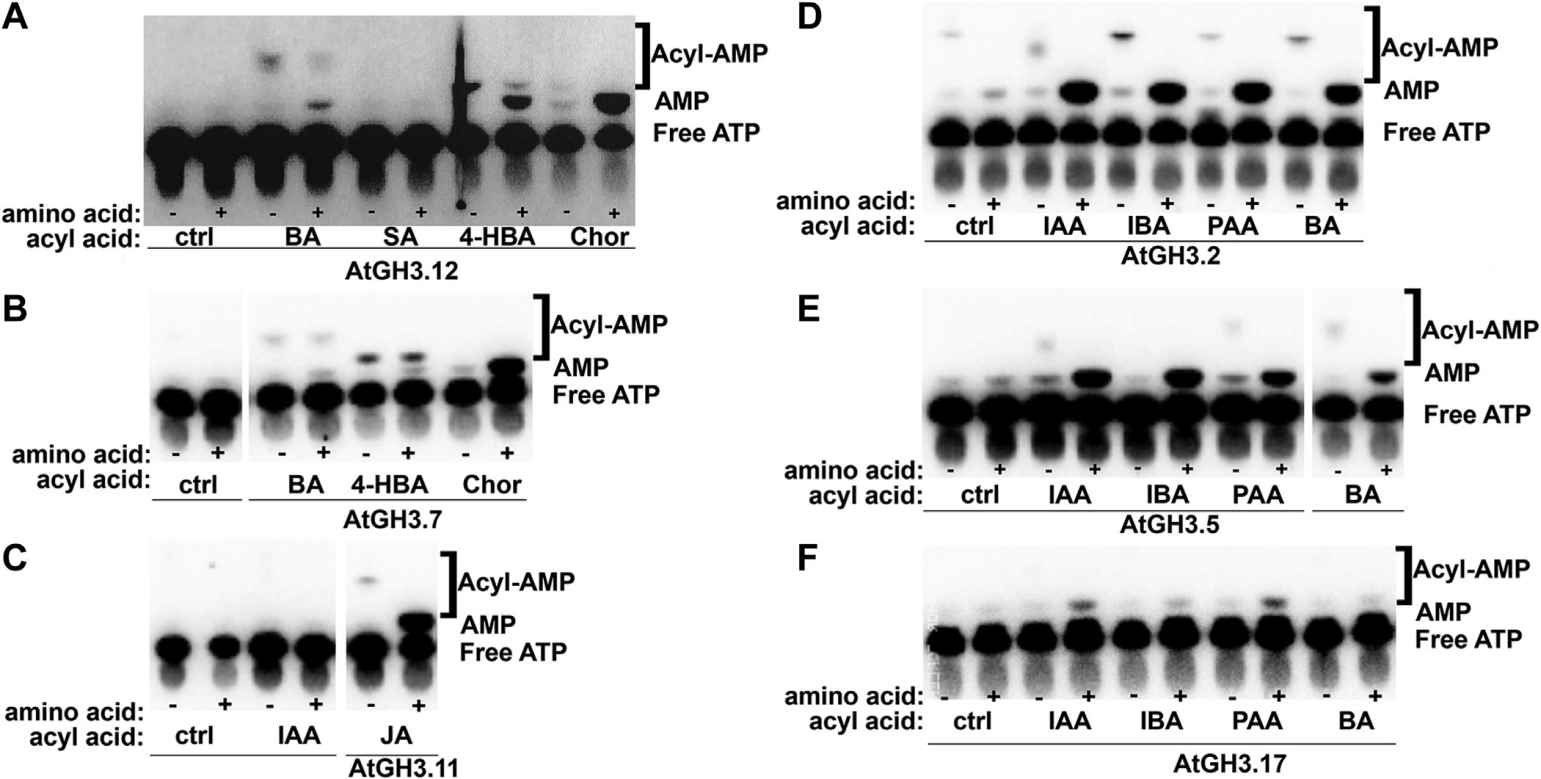
**Visualization of the first-half and full reactions of Arabidopsis GH3 proteins.** Reactions catalyzed by (*A*) AtGH3.12/PBS3, (*B*) AtGH3.7, (*C*) AtGH3.11/JAR1, (*D*) AtGH3.2/YDK2, (*E*) AtGH3.5/WES1, and (*F*) AtGH3.17/VAS2 with indicated acyl acid substrate and [α-^32^P]ATP in the absence (−) or presence (+) of amino acid substrate (glutamate: AtGH3.12/PBS3 and AtGH3.17/VAS2; cysteine: AtGH3.7; isoleucine: AtGH3.11/JAR1; aspartate: AtGH3.2/YDK2 and AtGH3.5/WES1) were analyzed by TLC. Positions of free radiolabeled ATP, AMP, and acyl-AMP are indicated in each panel.

To begin to unravel how GH3 proteins differentiate among acyl acid substrates, a TLC-based assay method for visualizing the first and second half-reactions of AtGH3.12/PBS3, AtGH3.7, AtGH3.11/JAR1, AtGH3.2/YDK2, AtGH3.5/WES1, and AtGH3.17/VAS2 with various acyl acid substrates was tested (Fig. 2). In these reactions, the acyl acid substrate, [α-^32^P]ATP, ± amino acid, and enzyme were mixed and the reaction quenched before being spotted on a TLC plate to separate formation of labeled acyl-AMP intermediate after the first half-reaction and release of free labeled AMP in the second half-reaction following addition of the amino acid. Use of [α-^32^P]ATP allowed for demonstration of acyl-adenylate intermediate formation in the absence of amino acid substrate, as well as conversion to AMP upon addition of amino acid. For AtGH3.12/PBS3 (Fig. 2*A*), incubation with the inhibitor SA confirmed no activity, whereas reactions using BA, 4-HBA, and chorismate produced acyl-AMP and AMP with the highest AMP observed with the cognate substrate pair chorismate and glutamate. A similar profile is observed with AtGH3.7 (Fig. 2*B*). With AtGH3.11/JAR1, products are observed only with JA as the acyl acid substrate (Fig. 2*C*). With the auxin-conjugating enzymes (AtGH3.2/YDK2, AtGH3.5/WES1, and AtGH3.17/VAS2), acyl-AMP and AMP products are observed with IAA, IBA, PAA, and BA (Fig. 2, *D–F*).

### Single-turnover analysis of GH3 protein-catalyzed reactions

**Figure 3:**
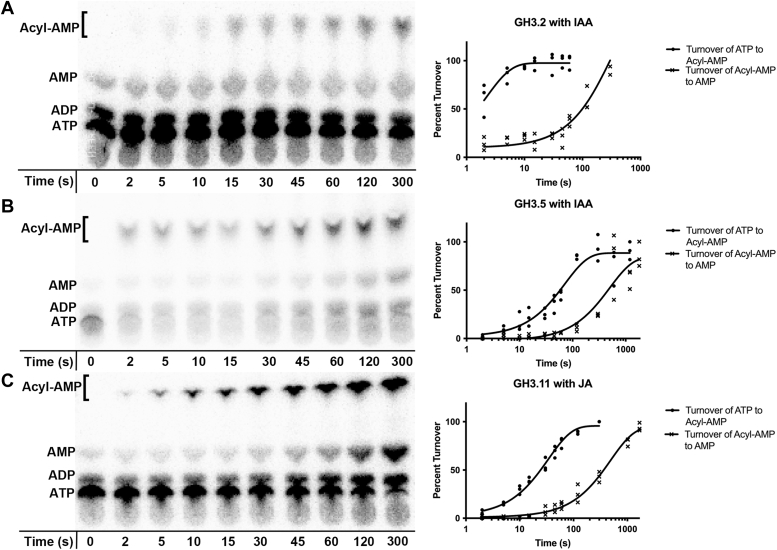
**Time course and single-turnover kinetics for acyl-AMP formation and AMP formation.** Time course of reactions catalyzed by (*A*) AtGH3.2/YDK2, (*B*) AtGH3.5/WES1, and (*C*) AtGH3.11/JAR1 were analyzed by TLC with positions of visualized radiolabeled nucleotides indicated (*left*). Plots of percent turnover *versus* time (*right*) show data as average ± SE (*n* = 3) with data fit as described in the Experimental procedures to determine rates of turnover from ATP to acyl-AMP (*k*_1_) and acyl-AMP to AMP (*k*_AMP_).

Using the TLC-based assay with [α-^32^P]ATP in the reaction mixture, the rates of the first half-reaction (*k*_1_) leading to the formation of the acyl-AMP intermediate for AtGH3.2/YDK2, AtGH3.5/WES1, and AtGH3.11/JAR1 were determined using single-turnover kinetics (Fig. 3). In these experiments, AtGH3.2/YDK2 (Fig. 3*A*) and AtGH3.5/WES1 (Fig. 3*B*) were assayed with IAA and AtGH3.11/JAR1 assayed with JA (Fig. 3*C*). Single-turnover kinetics were determined using a 10:1 M ratio of enzyme over substrate. Reactions were initiated by addition of enzyme with data fit to a first-order exponential to determine the rate of acyl-AMP formation (*k*_1_) for each of the three GH3 proteins with their acyl acid substrate (Table 2). In these reactions, not only was acyl-AMP accumulating over time, but AMP also accumulated in the first half-reaction. This accumulation was visible for all three GH3 proteins and a rate of AMP formation (*k*_AMP_) could be measured for each (Table 2). Using preferred substrates, AtGH3.2/YDK2 forms IAA-AMP at a rate of 21.5 min^−1^, while the rate of IAA-AMP formation with AtGH3.5/WES1 is 0.83 min^−1^ (Table 2). AtGH3.11 forms JA-AMP at a rate of 1.75 min^−1^. For each enzyme, the AMP formation rate (*k*_AMP_) was much slower than that of acyl-AMP formation (*k*_1_) (*i.e.*, 512-fold for AtGH3.2/YDK2; 13-fold for AtGH3.5/WES1; and 15-fold for AtGH3.11/JAR1).

**Table 2:** Single-turnover kinetic constants for Arabidopsis GH3 proteins

Protein	Rate constant	Acyl acid		
		IAA	PAA	BA
AtGH3.2/YDK2	*k*_1_	21.5 ± 2.8	9.06 ± 2.12	1.40 ± 0.39
	*k*_AMP_	0.042 ± 0.006	0.108 ± 0.018	0.072 ± 0.012
		IAA	PAA	BA
AtGH3.5/WES1	*k*_1_	0.834 ± 0.120	0.186 ± 0.030	0.186 ± 0.042
	*k*_AMP_	0.066 ± 0.012	0.042 ± 0.012	0.054 ± 0.006
		JA		
AtGH3.11/JAR1	*k*_1_	1.75 ± 0.12		
	*k*_AMP_	0.120 ± 0.006		

Rate constants *k*_1_ (rate of acyl-AMP formation; min^−1^) and *k*_AMP_ (AMP formation; min^−1^) were determined from time course experiments as described in the Experimental procedures. Rates are average ± SE (*n* = 3).

For comparison of preferred substrate *versus* non-preferred substrate, single-turnover experiments were also performed using BA and PAA as substrates for the two IAA-conjugating GH3 proteins (Table 2). With AtGH3.5/WES, BA-AMP and PAA-AMP form at comparable rates (0.186 min^−1^), while BA-AMP (1.40 min^−1^) forms at a slower rate than PAA-AMP (9.06 min^−1^) with AtGH3.2/YDK2. The rates of AMP release (*k*_AMP_) vary depending on the acyl acid substrate. For AtGH3.2/YDK2., AMP release with PAA (0.108 min^−1^) is nearly 2-fold faster than the rate with IAA (0.042 min^−1^) and 1.5 -fold faster than that determined with BA (0.072 min^−1^). The rates of AMP release with GH3.5 do not vary as much among the acyl acid substrates (0.066, 0.054, and 0.0042 min^−1^ for IAA, BA, and PAA, respectively). These rates indicate that AtGH3.2/YDK2 forms IAA-AMP intermediates faster than PAA-AMP with BA-AMP as the slowest reaction of the three substrates. With AtGH3.5/WES1, the rate of IAA-AMP is highest and nearly 5-fold faster than formation of PAA-AMP and BA-AMP. The partitioning between acyl-AMP formation and AMP release indicates that non-cognate substrates, like BA and PAA, tend to be released in higher proportion compared to IAA, the cognate substrate.

### Biochemical analysis of N- and C-terminal chimeras of AtGH3.2/YDK2 and AtGH3.5/WES1 suggest a role for conformational flexibility in substrate discrimination

Despite differences in catalytic efficiencies with various acyl acid substrates, AtGH3.2/YDK2 and AtGH3.5/WES1 use the same amino acid substrate (aspartate), and all the residues in their respective acyl acid binding pockets are nearly identical (25, 34). Multiple X-ray crystal structures of GH3 proteins imply rotation of the C-terminal domain by 180° between the two half reactions, (20, 21, 24, 25, 34, 46, 47, 48). In the first half-reaction (or open conformation), ATP and acyl acid substrate bind in the active site for formation of the acyl-AMP intermediate. This conformation has a solvent-accessible channel to allow for pyrophosphate release. In the second half reaction, the C-terminal domain rotates about the hinge loop, closes the active site, and repositions residues for the transferase reaction that forms the amino acid conjugate of the acyl acid substrate (34, 46, 48). The substrate profiles and reaction kinetics of the IAA-conjugating GH3 proteins may be related to differences in C-terminal movements.

**Figure 4:**
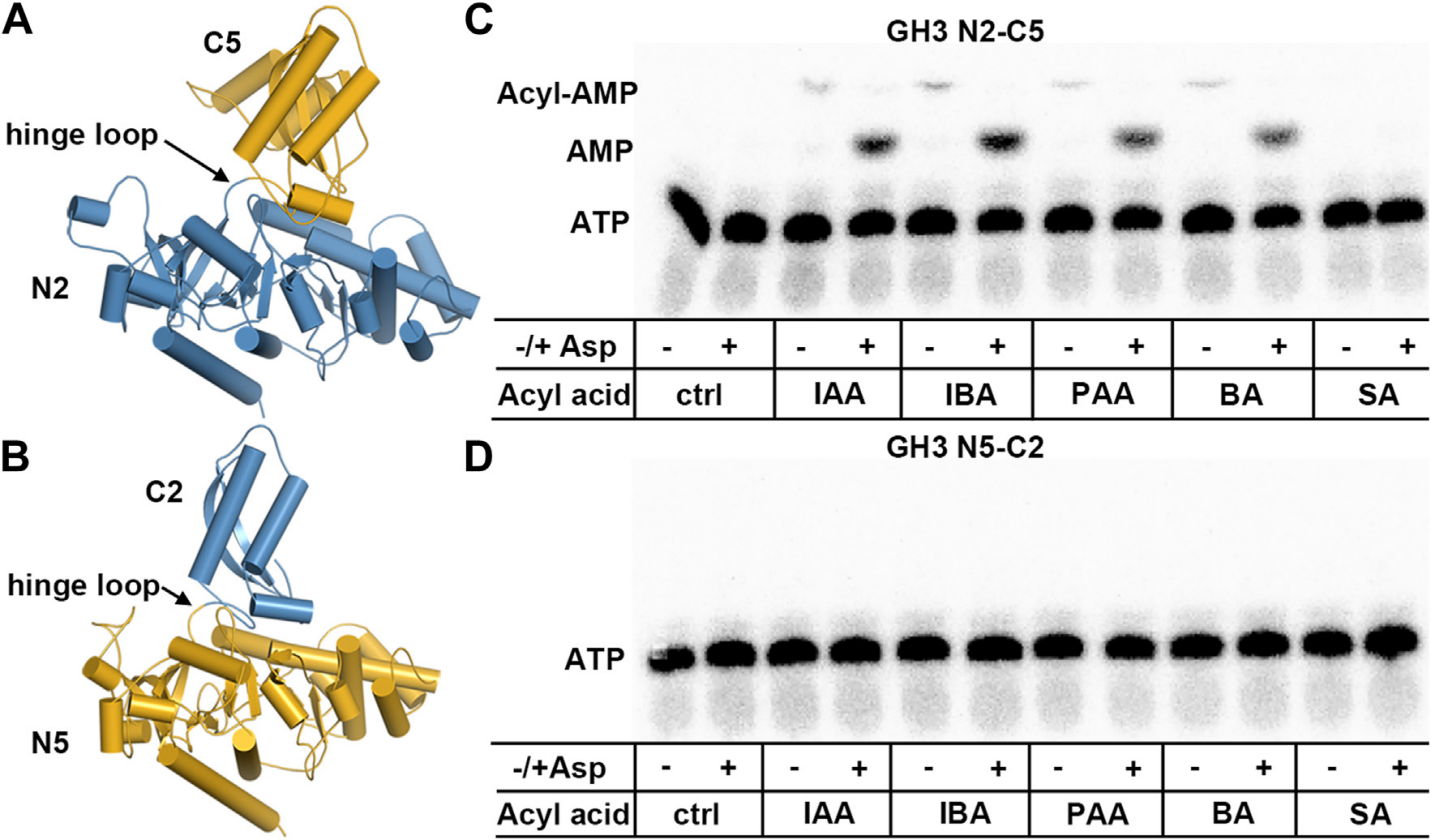
**Visualization of the first-half and full reactions of chimeric GH3 proteins.** Chimeric proteins (*A*) GH3-N2/C5 (N-terminal of AtGH3.2/YDK2 and C-terminal of AtGH3.5/WES1) and (*B*) GH3-N5/C2 (N- terminal of AtGH3.5/WES1 and C-terminal of AtGH3.2/YDK2) were generated to test the contribution of the flexible C-terminal domain on reaction kinetics. Structural models of each chimera are colored *blue* and *gold* to correspond to AtGH3.2/YDK2 and AtGH3.5/WES1, respectively. The location of the hinge loop near the active site is indicated. Reactions catalyzed by (*C*) GH3-N2/C5 and (*D*) GH3-N5/C2 with indicated acyl acid substrate and [α-^32^P]ATP in the absence (−) or presence (+) of amino acid aspartate were analyzed by TLC. Positions of free radiolabeled ATP, AMP, and acyl-AMP are indicated in each panel.

To ascertain if the C-terminal domain of GH3 proteins contributes to cognate *versus* non-cognate reaction kinetics, we generated chimeric proteins by swapping the C-terminal domains of AtGH3.2/YDK2 and AtGH3.5/WES1 (Fig. 4, *A* and *B* and S1). The N- and C-terminal domains are connected through the hinge loop region, which is where the site of the domain swap was introduced. The GH3-N2/C5 chimera retains the N-terminal domain of AtGH3.2/YDK2 (residues 1–442) and replaces the C-terminal domain with that of AtGH3.5/WES1 (residues 450–608). The GH3-N5/C2 chimera has the N-terminal domain of AtGH3.5/WES1 (residues 1–449) with the C-terminal domain of AtGH3.2/YDK2 (residues 443–549). Each protein was expressed and purified to levels comparable to both wild-type proteins and eluted from size-exclusion chromatography as monomeric forms, like the wild-type proteins.

The chimeric proteins were first assayed for activity using IAA, PAA, BA, or SA (as a non-substrate control) and [α-^32^P]ATP without and with aspartate (Fig. 4, *C* and *D*). The GH3-N5/C2 chimera had no detectable activity with any substrate; however, the GH3-N2/C5 chimera formed acyl-AMP for IAA, PAA, and BA in the absence of aspartate and formation of AMP in the presence of amino acid. Steady-state kinetic parameters for GH3-N5/C2 were determined using IAA, PAA, and BA as substrates (Table 3). In comparison to the two parent proteins (AtGH3.2/YDK2 having 5- to 10-fold higher catalytic efficiency with IAA *versus* PAA and BA and AtGH3.5/WES1 having comparable catalytic efficiencies for the three substrates - see introduction), the chimeric protein shows a change in substrate profile closer to that of AtGH3.5/WES1, which suggests that the C-terminal domain influences substrate discrimination without direct interaction with acyl acid substrates.

**Table 3:** Summary of steady-state kinetic parameters for chimeric protein GH3-N2/C5

Substrate	*k*_cat_ (min^−1^)	*K*_m_ (μM)	*k*_cat_/*K*_m_ M^−1^ s^−1^
IAA	13.5 ± 3.5	540 ± 17	417
PAA	26.0 ± 3.3	1500 ± 280	289
BA	17.1 ± 5.6	880 ± 68	323

Assays were performed as described in the Experimental procedures. Average values ± SE (*n* = 3) are shown for steady-state kinetic parameters.

## Discussion

GH3 acyl acid-amido synthetases help regulate active phytohormone concentrations within a plant cell using amino acid conjugation to generate either a storage or degradation form or an active form of a plant hormone (14, 15). Many acyl acid plant hormones, including the growth hormone IAA and the stress hormone JA are substrates of these proteins (8, 9, 10, 11, 12, 13, 14, 15, 16). Moreover, in Arabidopsis, the plant pathogen response hormone SA is synthesized from isochorismate by a GH3 protein (22, 23). Although most GH3 proteins are specific for a single hormone, several of these proteins have relaxed substrate preferences and can use similar acyl acids with almost equal catalytic efficiencies (25). In the case of non-specific GH3 proteins, such as AtGH3.5/WES1, the physiological roles of IAA-, PAA-, and BA-amino acid conjugates may greatly vary, so it seems that the protein either accepts multiple substrates as a way of controlling several metabolites simultaneously, or one is preferred over another for a more targeted physiological response. Despite intensive studies on the physiology, biochemistry, and three-dimensional structures of these proteins, our understanding of the mechanisms by which GH3 proteins select a substrate out of the milieu of acyl acids in the cell to form an amino acid conjugate escapes our current knowledge.

The two-step chemical mechanism of the GH3 proteins helps provide discrimination between cognate and non-cognate substrates (Fig. 1). Initial binding of acyl acid and ATP leads to the formation of an adenylated reaction intermediate and pyrophosphate (45), which undergoes the second transferase reaction in the presence of an amino acid. A previous study of AtGH3.12/PBS3 showed a difference in the specific activities for pyrophosphate release in the absence or presence of amino acid that differed between preferred and non-preferred substrates (24). With this GH3 protein, the pyrophosphate release activity was 26-fold higher with chorismate and glutamate than with chorismate alone; however, the specific activity of pyrophosphate formation with non-preferred substrates, like BA and 4-HBA, were generally comparable with or without amino acid (Table 1). This observation led us to assay AtGH3.7, AtGH3.11/JAR1, AtGH3.2/YDK2, AtGH3.5/WES1, and AtGH3.17/VAS2 for a pyrophosphate formation in the presence and absence of amino acid using preferred and non-preferred substrates (Table 1). AtGH3.11/JAR1 has an acyl acid binding site with a clear preference for oxylipin JA and no activity with IAA (Table 1), as described before (34). AtGH3.7, which shares a similar acyl acid substrate profile with AtGH3.12/PBS3, also had increased (9-fold) pyrophosphate release activity with chorismate and glutamate (Table 1). Similarly, the IAA-specific GH3 proteins AtGH3.2/YDK2 and AtGH3.17/VAS2 had higher ratios of activity with IAA in the presence of amino acid compared to non-preferred substrate; whereas AtGH3.5/WES1, which has more relaxed acyl acid preference had lower ratios of activity (Table 1).

These data suggest that binding of either a cognate (preferred) or non-cognate (non-preferred) acyl acid substrate and reaction with ATP leads to formation of an acyl-AMP intermediate, which is supported by TLC analysis of reactions using radiolabeled ATP (Fig. 2). For each GH3 protein assayed for pyrophosphate formation without addition of amino acid, there is some variation in activity with respect to acyl acid substrate (Table 1). In the case of AtGH3.11/JAR1, the acyl acid binding site structure is highly specific for the oxylipin JA, as reported previously (34); this explains the lack of activity with IAA. For AtGH3.5/WES1, which has a wider substrate profile than the other GH3 proteins, the activity with IAA, IBA, PAA, and BA all show more modest increased pyrophosphate release in the presence of amino acid. For AtGH3.12/PBS32, AtGH3.7, AtGH3.2/YDK2, and AtGH3.17/VAS2, the pyrophosphate release assay suggests that when a cognate acyl acid is adenylated in the presence of amino acid substrate, the second half-reaction rapidly occurs and results in the increased pyrophosphate release. In this case, the rate of synthesis from the acyl-AMP exceeds that of hydrolysis of the acyl-AMP. With non-cognate substrates, the reaction can continue but the ratio of pyrophosphate release is lower, which suggests that the rate of synthesis (continuing to the transferase reaction) *versus* hydrolysis of the non-cognate intermediate. For several of the GH3 protein-substrate combinations (*i.e.*, GH3.7 with BA; AtGH3.17/VAS2 with IBA or BA), pyrophosphate formation is equal with and without the addition of amino acid.

After developing a TLC assay to visualize the GH3 first half-reaction and full reaction (Fig. 2), we examined the question of substrate preference using single-turnover kinetics experiments (Fig. 3 and Table 2). These reactions measure the rate (*k*_1_) of the first half-reaction of two IAA-using GH3 proteins (AtGH3.2/YDK2 and AtGH3.5/WES1) with IAA, PAA, and BA and AtGH3.11/JAR1 with JA. The data show that AtGH3.2/YDK2 forms the cognate IAA-AMP intermediate at a rate much higher than with either PAA or BA as acyl acid substrates with a ratio of about 20:9:1 for IAA:PAA:BA (Table 2). From a physiological standpoint, it makes sense that the enzyme may have evolved the ability to generate PAA-amino acid conjugates since this hormone is able to interact with the TIR1/AFB auxin receptor that IAA binds and can regulate the same genes as free IAA (32). The physiological roles of PAA-Asp conjugates are not well understood in plants. For AtGH3.5/WES1, a generalist enzyme based on steady-state kinetics (25), the reaction rates were much slower than those of AtGH3.2/YDK2, but nonetheless there was a clear difference among the acyl acid substrates. IAA-AMP intermediates were formed at a rate that was about 4-fold faster than formation of either BA- or PAA-AMP intermediates. As noted above, AtGH3.11/JAR1 rapidly uses JA for formation of JA-AMP.

In this experiment, we noticed an accumulation of AMP in the first half-reaction for these proteins with these substrates in the absence of the amino acid (Fig. 3). Similar observations have been reported with other adenylating enzymes, such as the *Bacillus subtilis* biotin biosynthesis enzyme BioW (49). BioW functions as a pimeloyl-coenzyme A ligase and in similar experiments, there was an accumulation of AMP in the absence of the final product synthesis when a non-cognate substrate was assayed with the enzyme. In both BioW and the GH3 proteins, the adenylate intermediate is synthesized and then cleaved into free acyl acid and AMP, which is consistent with enzymatic proofreading to prevent non-cognate substrates from going through the complete reaction. The classic example of AMP accumulation with non-cognate substrates, a hallmark for enzymatic editing, or proofreading, is by tRNA synthetases (50, 51).

We calculated the rate of AMP accumulation in the first half-reaction of AtGH3.2/YDK2, AtGH3.5/WES1, and AtGH3.11/JAR1 (Table 2). For the IAA-conjugating enzymes, the rate of AMP formation (*k*_AMP_) was highest with PAA and lowest with IAA - a ratio of approximately 11:7:4 for PAA: BA: IAA, respectively, which is consistent with the cognate acyl-AMP as not being hydrolyzed as quickly as non-cognate intermediates. Once again, with AtGH3.5/WES1, the rates of AMP formation were more comparable with a ratio of 4:5:7 for PAA: BA: IAA, respectively (Table 2). Despite the rate of AMP accumulation being highest for IAA with AtGH3.5/WES1, there is more IAA-AMP being made compared to the degradation of the intermediate. Roughly, for every 13 IAA-AMP molecules formed, one will be hydrolyzed, whereas with BA and PAA about one in every four or five acyl-AMP molecules will be hydrolyzed to free acyl acid and AMP. This suggests that despite the generalist nature of this enzyme in steady-state kinetics assays, AtGH3.5/WES1 has a slight preference for IAA as a substrate, as reflected in the kinetics for the full reaction (25).

It is unclear if the acyl acid intermediate is hydrolyzed in the active site of the enzyme or in solution. One scenario is that the enzyme hydrolyzes the substrate using reactive residues in the active site, such as active site arginines in the case of BioW (52), with the free acyl acid and AMP diffusing away from the enzyme’s active site (49, 50, 51). An alternative hypothesis is that when a non-cognate substrate binds, the enzyme is unable to close, keeping the active site open for the adenylate to be released into solution where the acyl-AMP bond would be cleaved by a hydroxide ion (49). When the cognate substrate does bind, the enzyme closes and awaits the amino acid substrate for the transferase reaction to occur. Based on structural studies of the GH3 proteins, it is unlikely that there is a specific proofreading domain, as is the case for many of the tRNA synthetases (50, 51); instead, it is more likely that the enzyme edits for fidelity in the absence of such a domain.

To understand the structural component to pre-transfer substrate proofreading, we generated chimeras of two GH3 proteins (AtGH3.2/YDK2 and AtGH3.5/WES1) that used similar acyl acid substrates and the same amino acid substrate, aspartate (Fig. 4, *A* and *B* and S1). While the residues in contact with ligands in the structure of AtGH3.5/WES1 are conserved in AtGH3.2/YDK2 (24), the distal active site residues vary and the C-terminal domain of AtGH3.5/WES1 is larger than that of AtGH3.2/YDK2 ((24); Fig. S1). The GH3-N5/C2 enzyme was inactive, which suggests that the smaller C-terminal domain of AtGH3.2/YDK2 is insufficient for activity with the N-terminal domain of AtGH3.5/WES1; however, the GH3-N2/C5 enzyme was active in the adenylation reaction (Fig. 4, *C* and *D*). The specific impact of the GH3-N5/C2 chimeric construct on the reaction sequence is unclear and could impact binding of the acyl acid and/or ATP, which would abrogate the adenylation reaction. In contrast, steady-state kinetic analysis of GH3-N2/C5 indicated that the activity of the chimeric protein was closer to that of AtGH3.5/WES1 (Table 3), which suggests that the C-terminal domain influences substrate discrimination without direct interaction with acyl acid substrates.

**Figure 5:**
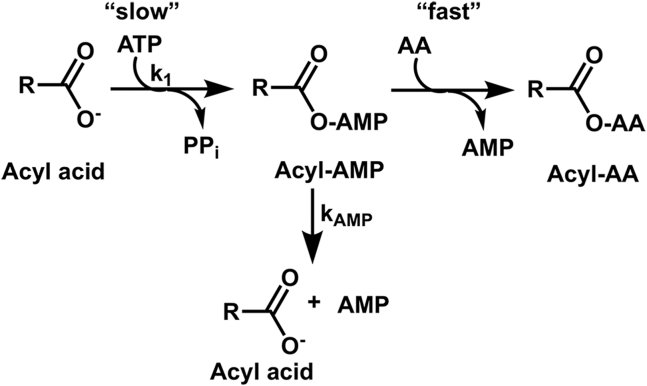
**GH3 reaction sequence and proofreading of adenylated intermediates.** In the first half-reaction, the acyl acid hormone is adenylated (*k*_1_); if the adenylated intermediate is non-cognate, the intermediate is hydrolyzed to AMP and acyl acid (*k*_AMP_). The second half-reaction is fast with cognate acyl-AMP intermediates and requires proper C-terminal domain movement.

Taken together, our data suggest that GH3 acyl acid amido synthetases in plants proofread substrates in the first half-reaction to ensure hormone substrate fidelity (Fig. 5). Movement of the C-terminal domain in the first half-reaction is critical to allow for the reaction to proceed (34, 46, 47, 48). Substrates bind the enzyme in the open conformation, and the transfer reaction takes place in the second half-reaction when the enzyme is in a closed conformation. The enzyme then reopens to release the conjugate and bind more substrates. Based on data generated here, the pivoting of the C-terminal domain to conform to the closed state occurs more quickly when the cognate substrate binds and is adenylated. When non-cognate substrates bind, the pivoting, or closing, of the active site is delayed, and the adenylated intermediate is degraded. This opening and closing of the enzyme allows the enzyme to proofread substrates before the transfer reaction to ensure the fidelity of the reaction, which in turn orchestrates the balance of active *versus* inactive hormones.

## Experimental procedures

### Protein expression and purification

AtGH3.2/YDK2, AtGH3.5/WES1, AtGH3.17/VAS2, AtGH3.12/PBS2, AtGH3.7, and AtGH3.11/JAR1 cloning, expression, and purification have been described previously (24, 25, 34). Codon-optimized fusion proteins GH3-N2/C5 (a chimeric construct with the N-terminal of AtGH3.2/YDK2 - residues 1–442 - and C-terminal of AtGH3.5/WES1 - residues 450–608; Fig. S1*A*) and GH3-N5/C2 (a chimeric construct with the N- terminal of AtGH3.5/WES1 - residues 1–449 - and C-terminal of AtGH3.2/YDK2 - residues 443–549; Fig. S1*B*) were synthesized (Genewiz) and cloned into the pET-28a expression vector, which were transformed into *E. coli* Rosetta2 (DE3) cells (Novagen). Cells were cultured in Terrific broth until *A*_600nm_ = 0.6 to 0.8 was obtained, at which time a final concentration of 0.75 mM isopropyl-β-D-1-thiogalactopyranoside (IPTG) was added to induce protein expression. After 16 h at 16 °C, cells were pelleted by centrifugation (5000*g*; 10 min) and resuspended in lysis buffer (50 mM Tris, pH 8.0, 500 mM NaCl, 20 mM imidazole, 10% (v/v) glycerol, and 1% (v/v) Tween-20). Following sonication, cell debris was removed by centrifugation (13,000*g*; 45 min). The resulting lysate was passed over a Ni^2+^-nitrilotriacetic acid (NTA) (Qiagen) column equilibrated in lysis buffer. The column was then washed (50 mM Tris, pH 8.0, 500 mM NaCl, 20 mM imidazole, and 10% (v/v) glycerol) and bound His-tagged protein eluted (50 mM Tris, pH 8.0, 500 mM NaCl, 250 mM imidazole, and 10% (v/v) glycerol). Eluted protein was further purified by size-exclusion chromatography using a Superdex-75 26/60 HiLoad ÄKTA FPLC size-exclusion column (GE Healthcare) equilibrated with 50 mM Tris (pH 8.0) and 100 mM NaCl. Samples were concentrated to ∼10 mg mL^−1^ using Centricon centrifugal filters with final protein concentration was determined by the Bradford method (Protein Assay, Bio-Rad) with bovine serum albumin as standard.

### Pyrophosphate-release assay

Pyrophosphate-release activity of AtGH3.2/YDK2, AtGH3.5/WES1, AtGH3.17/VAS2, AtGH3.12/PBS3, AtGH3.7, and AtGH3.11/JAR1 were determined using the pyrophosphate assay reagent kit (Sigma-Aldrich), as previously reported (24, 34), with ATP (2 mM), MgCl_2_ (2 mM), purified protein (10 μg), and various acyl acid substrates (5 mM IAA, IBA, PAA, and BA for AtGH3.2/YDK2, AtGH3.5/WES1, and AtGH3.17/VAS2; 5 mM IAA and JA for AtGH3.11/JAR1; 5 mM BA, 4-hydroxybenzoic acid (4-HBA), and chorismate for AtGH3.12/PBS3 and AtGH3.7) in the absence or presence of 10 mM aspartate (AtGH3.2/YDK2 and AtGH3.5/WES1), 10 mM glutamate (AtGH3.17/VAS2 and AtGH3.12/PBS3), 10 mM cysteine (AtGH3.7), or 10 mM isoleucine (AtGH3.11/JAR1). For assays with AtGH3.12/PBS3, commercially available chorismate (Sigma-Aldrich) was used instead of isochorismate, which is unstable and highly reactive under standard conditions. The commercially available pyrophosphate assay reagent kit uses a coupled enzyme system of fructose-6-phosphate kinase (0.5 units mL^−1^), aldolase (7.5 units mL^−1^), triosephosphate isomerase (10 units mL^−1^), and α-glycerophosphate dehydrogenase (5 units mL^−1^) in a defined buffer system (45 mM imidazole, pH7.4; citrate 5 mM, 0.10 mM EDTA, 2 mM Mg^2+^, 0.2 mM Mn^2+^, 0.02 mM Co^2+^, 0.8 mM NADH, 12 mM fructose-6-phosphate, 5 mg mL^−1^bovine serum albumin, and 5 mg mL^−1^ sugar stabilizer) for colorimetric determination of pyrophosphate formation. Assays were initiated by the addition of enzyme and were performed in triplicate at 25 °C using a Beckman DU800 UV/Vis spectrophotometer (A_340nm_) for colorimetric determination of pyrophosphate release according to manufacturer instructions with calculations accounting for 2 mol of NADH oxidized per mole of pyrophosphate consumed by the coupling system.

### Single-turnover kinetic thin-layer chromatography assays

For the initial TLC plate activity assays, individual reactions contained 50 mM Tris (pH 8.0), 1 mM MgCl_2_, 5 μM ATP with 1 μCi of [α-^32^P]ATP, 0.1 mM acyl acid substrate, and 50 μM protein. AtGH3.12/PBS3 was tested with BA, SA (a known inhibitor), 4-HBA, and chorismate. For AtGH3.7, the same acyl acid substrates except SA were used. AtGH3.11/JAR1 was tested with JA and IAA. AtGH3.2/YDK2, AtGH3.5/WES1, AtGH3.17/VAS2, and the two chimeric proteins, AtGH3 N2/C5, and AtGH3 N5/C2, were tested with IAA, IBA, PAA, and BA. To visualize the two half-reactions for these enzymes, 0.8 to 1 mM acyl acid substrate, 0.1 mM ATP, 1 μCi of [α-^32^P]ATP, in 50 mM Tris (pH 8.0) and 1 mM MgCl_2_ were used in the first half-reaction. Reactions (50 μl) were allowed to proceed for 0 to 5 min at 25 °C before they were quenched with formic acid (5 μl), after which 1 μl was spotted onto a cellulose thin-layer chromatography plate, which was developed in isobutyrate:ammonium hydroxide:water (66:1:33, v/v/v) (53). TLC plates were analyzed by phosphorimaging and quantified using Bio-Rad Quantity One software. Reactions were repeated in triplicate with rates determined by fitting the data to a one-phase decay equation (y = plateau∗(1-exp(-*k*∗x)), where y is percent turnover, plateau is the y value at infinite time, *k* the rate constant, and x is time) using GraphPad Prism software.

### Steady-state kinetic assays

Enzymatic activity of the GH3-N2/C5 and GH3-N5/C2 fusion proteins was evaluated spectrophotometrically using a coupled assay system that links AMP formation to the reactions of myokinase, pyruvate kinase, and lactate dehydrogenase, as previously described (24, 34, 45). Product formation is proportional to the rate of acyl acid conjugate formation with two NADH consumer for each molecule of conjugate. Standard reaction conditions were 20 mM Tris-HCl (pH 8.0), 1 mM MgCl_2_, 1 mM ATP, 5 mM aspartate, varied acyl acid (0–5 mM), 1 mM dithiothreitol, 2 mM phosphoenolpyruvate, 200 μM NADH, 4 units of rabbit muscle myokinase, 4 units of rabbit muscle pyruvate kinase, and 4 units of rabbit muscle lactate dehydrogenase in a total of 500 μl at 25 °C. Reactions were initiated by the addition of the enzyme (10 μg) and were conducted in triplicate with initial velocities determined using a Beckman DU800 UV/Vis spectrophotometer (A_340nm_). Data were fit to the Michaelis–Menten equation, *v* = *k*_cat_ [S]/(*K*_m_+[S), using GraphPad Prism.

## Data availability

All data are contained within the article.

## Supporting information

This article contains supporting information.

## Conflict of interest

J. M. J. is an associate editor of this journal. The other author declares that they have no conflicts of interest with the contents of this article.
