# Supplementary material for: Fidelity in plant hormone modifications catalyzed by Arabidopsis GH3 acyl acid amido synthetases
Source: J Biol Chem. 2024 May 28;300(7):107421. doi: 10.1016/j.jbc.2024.107421 (PMC11253546; doi:10.1016/j.jbc.2024.107421)
Supplement: Supplemental Figure S1 [file mmc1.docx]

**SUPPPORTING INFORMATION**

**Fidelity in Plant Hormone Modifications Catalyzed by GRETCHEN HAGEN 3 (GH3) Acyl Acid Amido Synthetases**

**Cynthia K. Holland^a,b^, Joseph M. Jez^b,*^**

Includes Supporting Figure S1

**Supplemental Figure S1. Amino acid sequence of GH3 fusion proteins.** (a) Sequence of the

GH3-N2/C5 fusion protein. The N-terminal sequence of AtGH3.2/YDK2 (residues 1-442) and the C-terminal sequence of AtGH3.5/WES1 (residues 450-608) are shown in blue and gold, respectively. (b) Sequence of the GH3-N5/C2 fusion protein. The N-terminal sequence of AtGH3.5/WES1 (residues 1-449) and the C-terminal sequence of AtGH3.2/YDK2 (residues 443-549) are shown in gold and blue, respectively.

(a) GH3-N2/C5 fusion protein

**MAVDSPLQSRMVSATTSEKDVKALKFIEEMTRNPDSVQEKVLGEILTRNSNTEYLKRFDLDGVVDRKTFKSKVPVVTYEDLKPEIQRISNGDCSPILSSHPITEFLTSSGTSAGERKLMPTIEEDLDRRQLLYSLLMPVMNLYVPGLDKGKGLYFLFVKSESKTSGGLPARPVLTSYYKSDHFKRRPYDPYNVYTSPNEAILCSDSSQSMYAQMLCGLLMRHEVLRLGAVFASGLLRAISFLQNNWKELARDISTGTLSSRIFDPAIKNRMSKILTKPDQELAEFLVGVCSQENWEGIITKIWPNTKYLDVIVTGAMAQYIPTLEYYSGGLPMACTMYASSESYFGINLKPMCKPSEVSYTIMPNMAYFEFLPHNHDGDGAAEASLDETSLVELANVEVGKEYELVITTYAGLYRYRVGDILRVTGFHNSAPQFKFIRRKNVVLSIDSDKTDEVELQNAVKNAVTHLVPFDASLSEYTSYADTSSIPGHYVLFWELCLDGNTPIPPSVFEDCCLAVEESFNTVYRQGRVSDKSIGPLEIKIVEPGTFDKLMDYAISLGASINQYKTPRCVKFAPIIELLNSRVVDSYFSPKCPKWVPGHKQWGSN**

(b) GH3-N5/C2 fusion protein

**MPEAPKKESLEVFDLTLDQKNKQKLQLIEELTSNADQVQRQVLEEILTRNADVEYLRRHDLNGRTDRETFKNIMPVITYEDIEPEINRIANGDKSPILSSKPISEFLTSSGTSGGERKLMPTIEEELDRRSLLYSLLMPVMSQFVPGLENGKGMYFLFIKSESKTPGGLPARPVLTSYYKSSHFKERPYDPYTNYTSPNETILCSDSYQSMYSQMLCGLCQHQEVLRVGAVFASGFIRAIKFLEKHWIELVRDIRTGTLSSLITDPSVREAVAKILKPSPKLADFVEFECKKSSWQGIITRLWPNTKYVDVIVTGTMSQYIPTLDYYSNGLPLVCTMYASSECYFGVNLRPLCKPSEVSYTLIPSMAYFEFLPVHRNNGVTNSINLPKALTEKEQQELVDLVDVKLGQEYELVVTTYAGLCRYRVGDLLRVTGFKNKAPQFSFICRKNVLLSVESDKTDEAELQKAVENASRLFAEQGTRVIEYTSYAETKTIPGHYVIYWELLGRDQSNALMSEEVMAKCCLEMEESLNSVYRQSRVADKSIGPLGDTCGTERYV**
